# Editorial: New roles for CD4^+^T cells in type 2 immune responses

**DOI:** 10.3389/fimmu.2023.1131819

**Published:** 2023-02-14

**Authors:** Nicholas van Panhuys, Hidehiro Yamane, Graham Le Gros

**Affiliations:** ^1^ Laboratory of Immunoregulation, Research Department, Sidra Medicine, Doha, Qatar; ^2^ Laboratory of Cellular and Molecular Biology, Center for Cancer Research, National Cancer Institute, National Institutes of Health, Bethesda, MD, United States; ^3^ Allergic and Parasitic Diseases Group, Malaghan Institute of Medical Research, Wellington, New Zealand

**Keywords:** type 2 immunity, Th2 (type-2) immune responses, Th2A cells, Tpath2, obesity, asthma, fibrosis, ILC2 - group 2 innate lymphoid cell

CD4^+^ T cell differentiation, originally described by Mossman and Coffman in their groundbreaking series of papers as a delineation of cells - based on their functional properties - into Th1 or Th2 subsets (1, 2), provided a plausible explanation for previous observations that described cellular and humoral immunity as being controlled by separate and distinct mechanisms (3). Subsequent studies into the type 2 inflammatory immune response controlled by Th2 cells described the complex network of cytokines and transcription factors needed to regulate B cell class switching and direct the production of specific immunoglobulins, as well as mediating the downstream activation of the innate immune system including eosinophils, basophils, macrophages and mast cells (4).

Initially, the role for CD4^+^ T cells in regulating and directing type 2 inflammatory responses was described in relation to Th2 differentiation and the induction of asthma and allergic responses on one side of the coin and the mediation of anti-parasitic responses on the other (5).

Recently, several new areas of research interest have opened up, exploring CD4^+^ control of type 2 inflammation associated responses for maintaining homeostasis in protecting against the onset of metabolic syndrome/T2D and muscle regeneration/wound repair. In parallel to these studies, the description of additional CD4^+^ T cell subsets and the contribution of specialized innate cells, as contributing to type II inflammation have also been recognized. This has led to the identification of Tfh, Th9, ILC2 and M2 macrophages, among others (6). Additionally, the role of CD4^+^ T cells in the production of the canonical Th2 associated cytokines, has yet to be adequately settled with the description of IL-4 production by Tfh (7) and the observation of a potential spatial and functional separation of IL-4 and IL-5/IL-13 producers during the immune response (8). As such, the role of the CD4^+^ T cell as an immune contributor to type II inflammation continues to evolve both in terms of its functional properties and also in relation to the diseases and tissues in which it plays a key role, under both homeostatic and inflammatory conditions.

## Asthma, allergy and the role of CD4^+^ T cells

Huang et al. describe the recent recognition of a subset of Th2 cells termed Th2A that are key regulators of the inflammatory immune response that drives the pathogenesis of allergic disease. Th2A cells have been characterised as a Th2 subset that is CD4^+^ CRTH2^+^ CD161^Hi^ HPGDS^+^ CD27^Lo^ CD49d^Hi^ ST2^Hi^ and have been used to both evaluate the severity of allergic disease and to determine the effectiveness of allergen specific immunotherapy. With decreases in Th2A cells being observed after treatment for food allergy with oral immunotherapy and further studies showing their utility as a potential biomarker for asthma and associated diseases.

The concept that asthma is a multifaceted disease driven by Th2 associated inflammatory responses in combination with input from additional Th subsets, including those previously characterised as being repressive of Th2 inflammation is explored in Luo et al. Here, the distinct spatial and temporal roles for Th1 and Th17 effectors in relation to Th2 cells is reviewed in the context of both acute and chronic asthmatic disease. Highlighting how the mutual crosstalk between multiple Th effector subsets may help explain the broad range of clinical endotypes encountered.

TGF-β1 has the potential to drive anti-inflammatory effects through Treg induction, whilst conversely it can also direct inflammatory responses through elicitation of Th9 or Th17 differentiation. In Musiol et al., the effects of the inducible ablation of *Tgfbr2* on CD4^+^ T cells during an allergic airway inflammatory response was assessed. Here, TGF-β signaling was found to exert the majority of its effects *via* the induction of Th2, Th9 and Th17 cells, with little effect observed on the presence of the Treg population. Coincident with these results cell populations present in atopic human subjects following natural allergen exposure showed a correlation between levels of TGF-β and the presence of pro-inflammatory cell types.

## Emerging roles for CD4^+^ in obesity, tissue repair and homeostasis

The role of type 2 inflammation in controlling obesity and regulating homeostasis to prevent the onset of metabolic disease has become an area of vast research interest. Schmidt et al. take a deep dive into the literature that surrounds the role of CD4^+^ T cells in obesity and metabolic disease and detail the metabolic processes that control differentiation of specific Th subsets in response to both environmental and inflammatory cues. Additionally, the authors review the role of CD4^+^ T cells during both obesity and malnutrition, and discuss the systemic effects of obesity on Th2 cells in a variety of disease settings.

In Kokubo et al., the authors review the role of both conventional and pathogenic Th2 cells in mediating those responses that occur in order to mediate the repair of tissue damage induced during inflammation and describe the necessity of a tight control over the response evoked in order to prevent excessive or chronic fibrosis. Additionally, a detailed description of the recently described Tpath2 cells (alternately referred to as Th2A or peTh2) is provided and their role in the management of pathogenesis, fibrosis and tissue repair is described.

## Novel technical resource

GATA3 has a central role in the type 2 immune response, as the master transcriptional regulator required for the development of ILC2 and differentiation of Th2 cells. Gurram et al. develop and characterize the use of *Gata3* ^ZsG^ and *Gata3* ^ZsG-fl^ alleles as novel reporters for studying Th2 cells and ILC2 functionality. Here, insertion of a ZsGreen reporter at the translation initiation site of *Gata3* allowed accurate *in vivo* tracking of *Gata3* expression and the combination of floxed *Gata3* ^ZsG-fl^ with a tamoxifen inducible Cre allowed for the inducible deletion and tracking of both ILC2 and Th2 cells during an *in vivo* response. With results indicating GATA3 is dispensable for regulating its own expression in mature ILC2’s and suggesting that GATA3-deficient “ILC2s” are more stable under *in vivo* conditions.

## General summary

In this Research Topic, we collected articles on emerging and novel CD4^+^ driven-responses that add to our knowledge on the diverse nature of type 2 immunity and inflammation, beyond the originally described concepts of Th2-regulated allergic/parasitic diseases. All together, these recent insights shed light on the broad functional relevance of CD4^+^ T cells in mediating those type 2 immune responses that encompass more than just the induction of peripheral Th2-associated humoral immunity.

## Author contributions

All authors listed have made a substantial, direct, and intellectual contribution to the work and approved it for publication.
